# A Reverse-Transcription Loop-Mediated Isothermal Amplification Technique to Detect Tomato Mottle Mosaic Virus, an Emerging Tobamovirus

**DOI:** 10.3390/v15081688

**Published:** 2023-08-03

**Authors:** Kan Kimura, Akio Miyazaki, Takumi Suzuki, Toya Yamamoto, Yugo Kitazawa, Kensaku Maejima, Shigetou Namba, Yasuyuki Yamaji

**Affiliations:** Department of Agricultural and Environmental Biology, Graduate School of Agricultural and Life Sciences, The University of Tokyo, Tokyo 113-8657, Japan

**Keywords:** plant virus, RT-LAMP, tobamovirus, ToMMV, toothpick sampling

## Abstract

Tomato mottle mosaic virus (ToMMV) is an emerging seed-transmissible tobamovirus that infects tomato and pepper. Since the first report in 2013 in Mexico, ToMMV has spread worldwide, posing a serious threat to the production of both crops. To prevent the spread of this virus, early and accurate detection of infection is required. In this study, we developed a detection method for ToMMV based on reverse-transcription loop-mediated isothermal amplification (RT-LAMP). A LAMP primer set was designed to target the genomic region spanning the movement protein and coat protein genes, which is a highly conserved sequence unique to ToMMV. This RT-LAMP detection method achieved 10-fold higher sensitivity than conventional RT-polymerase chain reaction methods and obtained high specificity without false positives for closely related tobamoviruses or healthy tomato plants. This method can detect ToMMV within 30 min of direct sampling of an infected tomato leaf using a toothpick and therefore does not require RNA purification. Given its high sensitivity, specificity, simplicity, and rapidity, the RT-LAMP method developed in this study is expected to be valuable for point-of-care testing in field surveys and for large-scale testing.

## 1. Introduction

Plant diseases are among the major threats to food supply worldwide. An estimated 20–30% of annual global crop production is lost due to plant pathogens and pests [1]. Emerging pathogens are particularly likely to cause severe damage to crops because effective control measures such as pesticides and resistant cultivars have not yet been prepared. Among various types of pathogens, viruses have been estimated to account for almost half of all emerging plant diseases [2]; for example, 45 novel viruses and satellite viruses for tomato emerged from 2011 to 2020 [3]. In the absence of antiviral chemicals and cultivars resistant to emerging viruses, an alternate strategy is the prompt identification of infected plants, followed by their removal and interception of the infection routes. Thus, it is essential to establish suitable detection methods for each emerging virus.

A variety of methods have been developed to diagnose viral infections, among which polymerase chain reaction (PCR) and real-time PCR are the most commonly used due to their high reliability. However, PCR-based methods require specialized equipment such as a thermal cycler or real-time fluorescence detection system and are time-consuming. Therefore, there is a growing need for alternative virus detection methods that are rapid and easily accessible. Recently, loop-mediated isothermal amplification (LAMP) [4], which uses four to six different primers that recognize six to eight regions of the target sequence, has been recognized as an alternative for overcoming the disadvantages of PCR. The LAMP system enables rapid, specific, and sensitive DNA amplification under isothermal conditions (approximately 60–65 °C) without specialized equipment, and applies to RNA templates through the addition of reverse transcriptase (RTase). The LAMP reaction is usually sufficiently robust to occur on crude samples that may be prepared by direct toothpick sampling from leaves [5], avoiding the need for laborious DNA/RNA purification. These features allow for point-of-care testing in the field, making LAMP an attractive diagnostic method for viral infections.

The *Tobamovirus* genus (family *Virgaviridae*) is a major group of viruses that cause serious damage to crops. Tobamoviruses have a single-stranded positive-sense RNA genome that is wrapped in a rod-shaped particle. The genome contains four open reading frames (ORFs 1–4): two RNA replicases, a movement protein (MP), and a coat protein (CP). The threat posed by tobamoviruses is attributed to their high transmissibility and particle stability. Tobamoviruses can be easily transmitted mechanically through plant–plant contact, agricultural implement contamination, and human or bumblebee contact [6,7]; they can also be transmitted through seeds via attachment to the seed surface, despite low transmission rates [8,9]. The high stability of viral particles enables them to persist in the environment, such as soil and water, while retaining their infectious properties [10,11].

Recently, two emerging tobamovirus species were reported: Tomato mottle mosaic virus (ToMMV) [12] and Tomato brown rugose fruit virus (ToBRFV) [13]. Both viruses mainly infect tomato, which is among the most important vegetable crops worldwide, with production exceeding 184 million tons in 2020 [14]. ToMMV infection induces mosaic, mottle, narrowing, and crinkling symptoms on tomato plant leaves, as well as fruit necrosis [15,16], leading to stunted growth and reduced fruit yield. The virus was initially found in greenhouse-grown tomatoes in Mexico in 2013 [12], and subsequently detected in China [17], the USA [18], Israel [19], Spain [20], Brazil [21], Czechia [22], Mauritius [23], the Netherlands [24], and France [25]. Tomato (*Solanum lycopersicum* L.) and pepper (*Capsicum annuum* L.) [12,17] are the major hosts of ToMMV, and chickpea (*Cicer arietinum* L.) [26], eggplant (*Solanum melongena* L.) [27], and pea (*Pisum sativum* L.) [28] have also been reported as natural hosts. Under experimental conditions, plants in the families *Solanaceae*, *Brassicaceae*, and *Amaranthaceae* have been shown to be susceptible to systemic infection by ToMMV [15,29].

To protect tomato from tobamovirus infection, the resistance genes *Tm-1*, *Tm-2*, and *Tm-2*^2^ have been widely incorporated into many commercial cultivars, protecting the most common tobamoviruses, Tobacco mosaic virus (TMV) and Tomato mosaic virus (ToMV). In particular, *Tm-2*^2^ has been applied for many years for its high durability [30,31]. However, ToBRFV has been reported to overcome resistance genes [32], and several lines of evidence indicate that ToMMV infects ToMV-resistant tomato cultivars with a certain frequency [15,33]. Therefore, early detection of viral infection is the key to managing these emerging viruses.

Previous studies have reported the successful development of reverse-transcription (RT)-LAMP methods for detecting ToBRFV with high sensitivity [34,35,36]. However, an RT-LAMP method for ToMMV has not yet been reported. Therefore, in this study, we developed an RT-LAMP-based detection system for ToMMV. We designed a primer set, determined the optimal reaction conditions, and evaluated the sensitivity and specificity of the system. Finally, we detected ToMMV using RT-LAMP following direct toothpick sampling from tomato leaves, which demonstrated the applicability of the newly developed system for on-site diagnosis and will contribute to the rapid control of this emerging tobamovirus.

## 2. Materials and Methods

### 2.1. Virus Isolates and Plant Materials

We used ToMMV isolate PV-1267 (GenBank accession no. MW582804), which originated from tomato in California in 2016 and was provided by the Deutsche Sammlung von Mikroorganismen und Zellkulturen (DSMZ, Braunschweig, Germany). We selected five closely related tobamoviruses, TMV, ToMV, Rehmannia mosaic virus (RheMV), ToBRFV, and Paprika mild mottle virus (PaMMV), as non-targets to test cross-reactivity. The OM TMV isolate (GenBank accession no. D78608) was collected from tobacco [37]; the L ToMV isolate (GenBank accession no. X02144) was collected from tomato; the Japanese RheMV isolate (GenBank accession no. AB628188) was sampled from chili pepper and kindly provided by the National Agriculture and Food Research Organization (NARO, Ibaraki, Japan); the Israeli ToBRFV isolate PV-1241 (GenBank accession no. MZ202349) was sampled from tomato and provided by DSMZ; and the Japanese PaMMV isolate (GenBank accession no. AB089381) was sampled from sweet pepper and provided by NARO. These viruses were mechanically inoculated onto the upper unfolded leaves of 4-week-old *Nicotiana benthamiana* plants; the uppermost leaves were collected at 7 days post-inoculation (dpi) and used for RNA extraction and subsequent inoculation. For tomato plant inoculation, virus-infected *N. benthamiana* leaves were ground in an inoculation buffer consisting of 0.1 M phosphate buffer (pH 7.0) and 0.1 M dithiothreitol. The inocula were mechanically inoculated onto the fourth leaves of the “Roma VF” tomato cultivar at the 4–5-leaf stage. The uppermost leaves (fifth or sixth leaves) were collected at 7 dpi. An untreated tomato plant was also prepared as a negative control. All plants were grown in a greenhouse at 25 °C under natural light conditions.

### 2.2. Total RNA Extraction

Total RNA was extracted from 20 mg of fresh leaves of *N. benthamiana* and tomato plants using the FavorPrep Plant Total RNA Purification Mini Kit (Favorgen, Pingtung, Taiwan) according to the manufacturer’s instructions, with the following modifications: 3-mercapto-1,2-propanediol was added to FARB buffer instead of β-mercaptoethanol, and each centrifugation step was conducted at 10,000× *g*. Total RNA was finally eluted with distilled water (DW) and quantified using a NanoDrop One spectrophotometer (Thermo Fisher Scientific, Waltham, MA, USA) and stored at −30 °C.

### 2.3. RT-PCR

RT-PCR was performed using Tob-Uni1/2 [38] and ToMMV-F/R [15], with amplicon sizes of approximately 800 and 289 bp, as tobamovirus-universal and ToMMV-specific primer pairs, respectively (Table 1). To confirm viral infection, we performed a two-step RT-PCR. cDNA was synthesized from 50 ng of total RNA using the High-Capacity cDNA Reverse-Transcription Kit (Applied Biosystems, Waltham, MA, USA) using random primers, and the resulting cDNA was amplified by PCR using *LA Taq* DNA polymerase (TaKaRa, Tokyo, Japan) with Tob-Uni1/2 according to the manufacturer’s instructions. The reaction conditions were as follows: 94 °C for 1 min; 35 cycles of denaturation at 98 °C for 10 s, annealing at 55 °C for 1 min, and elongation at 68 °C for 1 min; and final elongation at 68 °C for 7 min. For sensitivity comparison with RT-LAMP, one-step RT-PCR was performed using 50 ng of total RNA as described previously [39], with the following modifications: RT at 55 °C, 40 cycles of denaturation, elongation for 30 s for ToMMV-F/R and 1 min for Tob-Uni1/2, and final elongation for 5 min. RT-PCR amplicons were analyzed by electrophoresis on 2% (*w*/*v*) agarose gels in TAE buffer, with the 100-bp DNA ladder (New England Biolabs, Ipswich, MA, USA).

### 2.4. RT-LAMP Primer Design

RT-LAMP primers were designed based on the sequence of the ToMMV isolate YYMLJ (GenBank accession no. KR824950) using the PrimerExplorer V5 software (https://primerexplorer.jp/, accessed on 1 March 2022) with the default options.

### 2.5. RT-LAMP

RT-LAMP reactions were performed using an RNA amplification reagent kit (code no. NE6051) purchased from Nippon Gene (Tokyo, Japan) according to the manufacturer’s instructions, and monitored with the Genie I instrument (OptiGene, Horsham, UK). The reaction mixture contained 0.2 µM of forward outer primer (FOP) and backward outer primer (BOP), 1.6 µM of forward inner primer (FIP) and backward inner primer (BIP), and 0.8 µM of forward loop primer (LF) and backward loop primer (LB). The mixture was incubated at 63 °C for 45–120 min for the amplification reaction and then incubated at 80 °C for 5 min to inactivate enzymes. Positive reactions were visualized by a fluorescent detection reagent included in the reaction mixture, which emits green fluorescence to indicate pyrophosphate ion, the by-product of the chain elongation. Real-time amplification data monitored by the Genie I instrument was output graphically as either fluorescence or its increasing ratio against time, which is calculated by differentiating the fluorescence value. The peak time of the ratio was regarded as the detection time because it depends on the amount of virus in the mixture and can be interpreted as C_t_ or C_q_ in a real-time PCR assay. The endpoint amplification was also detected by the naked eye and a digital camera under natural or ultraviolet light.

### 2.6. Direct Sampling of ToMMV from Infected Tomato Leaves

As an alternative to RNA extraction, we obtained samples directly using wooden toothpicks as follows. An upper (fifth or sixth leaf) tomato leaf was placed on paper and pricked vertically in one or three random areas using a toothpick without penetration, and then the toothpick was dipped into the RT-LAMP reaction mixture. Three ToMMV-infected tomato plants were tested at 7 dpi as biological replicates. Total RNA was extracted from the pricked leaves and a 10-fold dilution series was also subjected to RT-LAMP, as described above. TMV-, ToMV-, RheMV-, and ToBRFV-infected tomato plants were also subjected to the assay at 7 dpi by obtaining three pricking samples with two biological replicates. A healthy tomato plant was used as a negative control.

## 3. Results

### 3.1. RT-LAMP Primer Design and Selection

**Figure 1:**
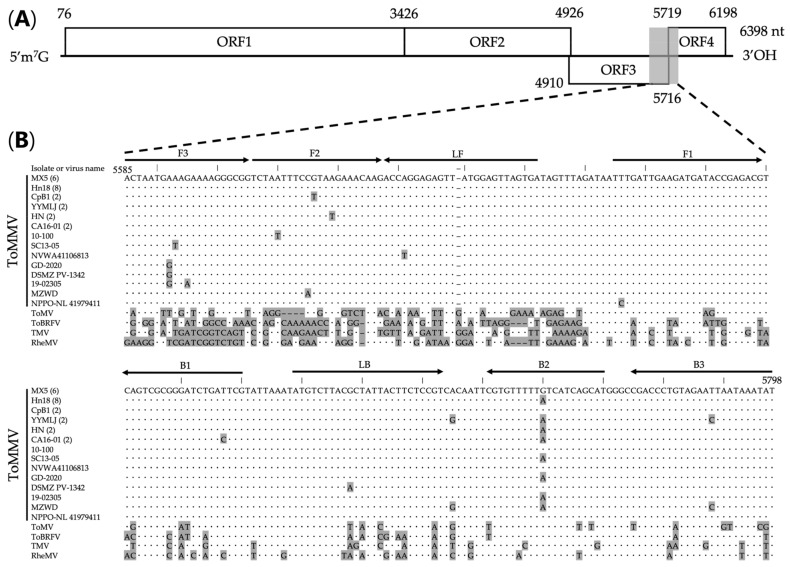
The design of reverse-transcription loop-mediated isothermal amplification (RT-LAMP) primer for the specific detection of Tomato mottle mosaic virus (ToMMV). (**A**) The ToMMV genome and region amplified by the RT-LAMP primer set designed in this study. (**B**) Alignment sequences of ToMMV (5585–5798 nt) and closely related tobamoviruses: Tobacco mosaic virus (TMV), Tomato mosaic virus (ToMV), Rehmannia mosaic virus (RheMV), and Tomato brown rugose fruit virus (ToBRFV). Numbers in parentheses after ToMMV isolate names indicate the numbers of reported isolates with the same sequence in the amplified region. The positions and directions of the primers are indicated by arrows. Nucleotides that differ from those of the MX5 isolate are shaded; identical nucleotides are indicated by dots. Dashes indicate nucleotide deletions. The sequence of each primer was identical to that of MX5, except for one nucleotide in B2.

We designed 42 primer sets across the whole genome of the ToMMV isolate YYMLJ. Because loop primers can improve amplification efficiency [40], we selected six primer sets in which both loop primers were designed. Among these, we selected one primer set targeting unique sequences conserved among ToMMV isolates available in the National Center for Biotechnology Information (NCBI) nucleotide sequence database (accessed on 13 April 2023; Table S1). The primer set was designed in the genomic region spanning the MP and CP genes; the nucleotide sequence of MP showed >98% sequence identity with ToMMV and <80% with close relatives [41]. The primers were modified by replacing minor single-nucleotide polymorphism alleles with major ones, and are shown in Table 1 and Figure 1.

### 3.2. Determination of Reaction Conditions

**Figure 2:**
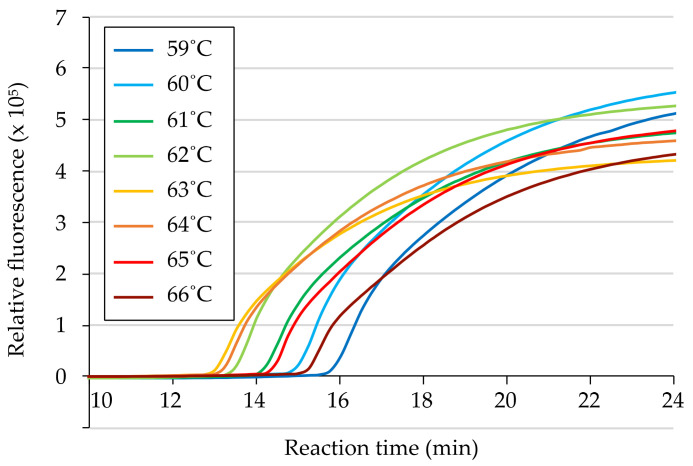
Determination of the optimal RT-LAMP reaction temperature. RT-LAMP amplification curves were obtained using the primers listed in Table 1, and 50 ng of total RNA was extracted from a ToMMV-infected *Nicotiana benthamiana* as a template. RT-LAMP reactions were conducted at incubation temperatures from 59 to 66 °C, at 1 °C intervals, for 45 min.

To determine the optimal thermal condition for the primer set, RT-LAMP reactions were conducted at a range of temperatures from 59 °C to 66 °C, at 1 °C intervals, using 50 ng of total RNA from a ToMMV-infected *N. benthamiana* leaf as a template. The detection times, identified as the peak time in the fluorescence ratio graph, were compared; positive reactions were observed under all thermal conditions within 17 min, with the earliest positive reaction obtained at approximately 13 min at a temperature of 63 °C (Figure 2). Based on these results, we selected 63 °C as the optimal temperature for the primer set, and subsequent RT-LAMP reactions were performed at 63 °C.

### 3.3. Specificity of ToMMV Detection by RT-LAMP

**Figure 3:**
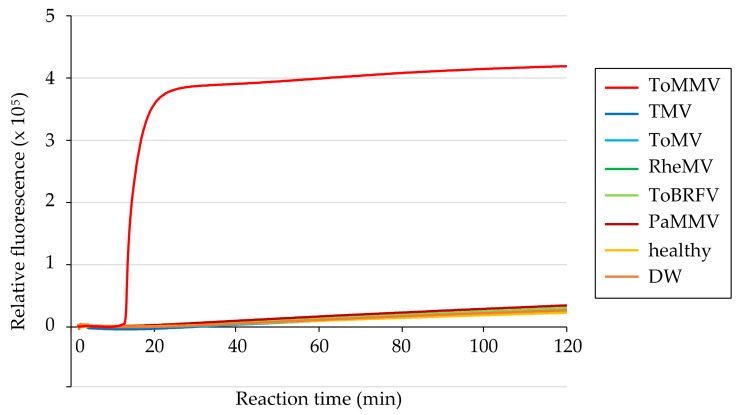
Specificity of the RT-LAMP primer set for detecting ToMMV using 50 ng of total RNA extracted from *N. benthamiana* plants infected with ToMMV, TMV, ToMV, RheMV, ToBRFV, or Paprika mild mottle virus (PaMMV) as templates. Total RNA from a healthy tomato plant (healthy) and distilled water (DW) were used as negative controls. RT-LAMP reactions were conducted at 63 °C for 120 min.

The specificity of the primer set was tested using 50 ng of total RNA from a healthy “Roma VF” tomato leaf and *N. benthamiana* leaves infected with non-target tobamoviruses (TMV, ToMV, RheMV, ToBRFV, or PaMMV). After 120 min of reaction, DNA amplification was not observed in any samples as in DW (non-template negative control), whereas a positive reaction was confirmed within 20 min for total RNA from the ToMMV-infected plant (Figure 3), which was sufficiently rapid for detection. These results were consistent across three replicates, demonstrating the high specificity of the RT-LAMP system.

### 3.4. Relative Sensitivity of ToMMV Detection by RT-LAMP

**Figure 4:**
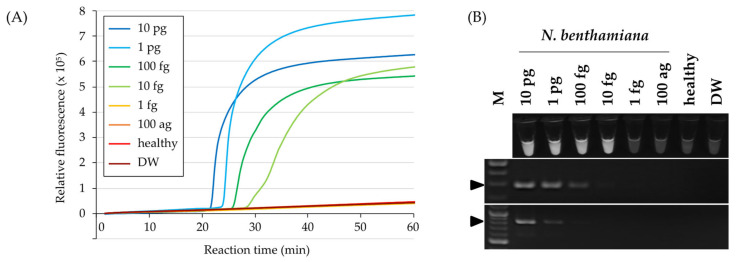
Comparison of relative sensitivity between RT-LAMP and one-step RT-polymerase chain reaction (PCR) for ToMMV detection. (**A**) RT-LAMP amplification curves were obtained using a 10-fold dilution series of total RNA (100 ag to 10 pg) extracted from a ToMMV-infected *N. benthamiana* plant as a template. Total RNA from a healthy tomato plant (healthy) and distilled water (DW) were used as negative controls. RT-LAMP reactions were conducted at 63 °C for 60 min. (**B**) Visual detection of RT-LAMP (upper) and one-step RT-PCR with primer pairs ToMMV-F/R (middle) or Tob-Uni1/2 (lower) under ultraviolet light. “M” indicates the 100-bp DNA ladder (New England Biolabs, Ipswich, MA, USA). Arrows indicate amplicon bands.

The sensitivity of RT-LAMP was compared with that of one-step RT-PCR using the same 10-fold dilution series of total RNA extracted from a ToMMV-infected *N. benthamiana* plant. For RT-LAMP, positive reactions were observed for 10 fg to 10 pg template RNA (Figure 4A), but not for 100 ag and 1 fg template RNA or negative controls. By contrast, amplifications were observed from 100 fg to 10 pg for ToMMV-specific RT-PCR, and from 1 pg to 10 pg for tobamovirus-universal RT-PCR (Figure 4B). Consistent results were observed in three independent replicates, indicating that the developed RT-LAMP method was 10- and 100-fold more sensitive to ToMMV than the ToMMV-specific and tobamovirus-universal RT-PCR methods, respectively.

### 3.5. Evaluation of the Direct Sampling Method from Infected Tomato Leaves

**Figure 5:**
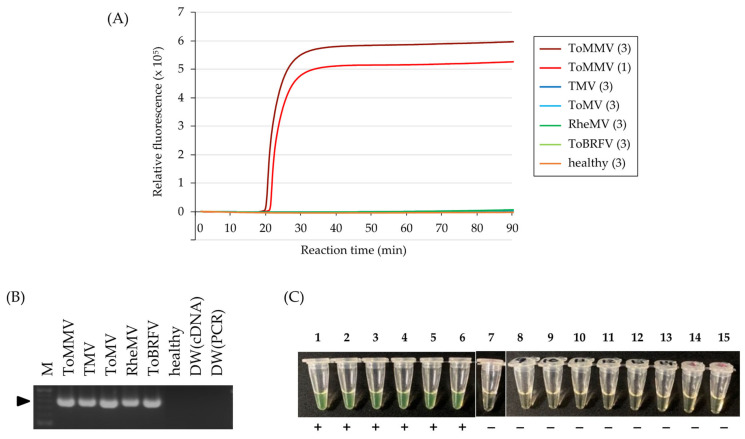
Direct detection of ToMMV from infected tomato leaves sampled using a wooden toothpick. RT-LAMP reactions were conducted at 63 °C for 90 min. (**A**) RT-LAMP amplification curves were constructed using toothpick-pricked leaf samples infected with ToMMV, TMV, ToMV, RheMV, or ToBRFV as templates. A non-treated healthy tomato leaf sample (healthy) was used as a negative control. Numbers in parentheses indicate the number of leaf pricks administered. Only one replication is represented for each virus. (**B**) Agarose gel electrophoresis of RT-PCR amplicons using the primer pair Tob-Uni1/2 with total RNA (50 ng) extracted from the tomato leaves used in the RT-LAMP reaction as templates. “M” indicates the 100-bp DNA ladder (New England Biolabs, Ipswich, MA, USA). “DW (cDNA)” and “DW (PCR)” indicate non-template negative controls for cDNA synthesis and PCR steps of two-step RT-PCR. An arrow indicates an amplicon band. (**C**) Fluorescence detection of RT-LAMP amplicons under natural light. Each template was as follows. (1, 2), (3, 4), and (5, 6) represent three independent ToMMV-infected tomato plant leaves. (7) represents a non-treated healthy tomato leaf sample. (8, 9), (10, 11), (12, 13), and (14, 15) represent TMV-, ToMV-, RheMV-, and ToBRFV-infected tomato leaves sampled from two independent tomato plants. (1), (3), and (5) were pricked once; all other samples were pricked three times. “+” indicates positive and “-” indicates negative.

For on-site detection of ToMMV infection in tomato plants, we attempted to omit the RNA purification process by performing direct sampling using a wooden toothpick. A ToMMV-infected tomato leaf was pricked with a toothpick one or three times, and the toothpick was then dipped into the RT-LAMP reaction mixture. Positive reactions were observed within 23 min in both prick counts, with slightly faster detection achieved for the three-prick method than the single-prick method (Figure 5A). This result was replicated three times. No amplification was observed for a healthy tomato plant, even using the three-prick method. We also confirmed that ToMMV was specifically detected using this sampling method in the tomato cultivars “Momotaro” and “Brandywine black” (Figure S1). These tests were also conducted for tomato leaves infected with TMV, ToMV, RheMV, or ToBRFV, whose single infection was verified by RT-PCR (Figure 5B) and Sanger sequencing. Notably, no cross-reactive amplification was observed in the RT-LAMP reaction for 90 min (Figure 5C). These results indicate the applicability of this toothpick sampling method to detect ToMMV infections in tomato plants.

**Figure 6:**
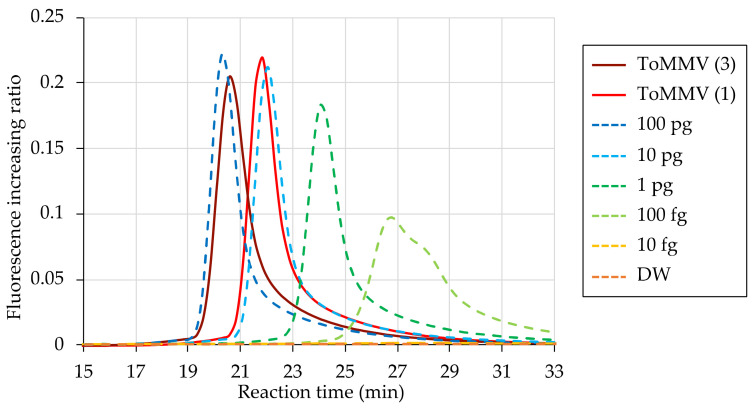
Evaluation of direct detection of ToMMV from infected leaves using a toothpick compared with that based on total RNA extracted from an infected plant. Fluorescence ratio data are based on toothpick sampling (solid lines) and a 10-fold dilution series of total RNA (dashed lines) used as templates for the RT-LAMP method. Numbers in parentheses after the sample name indicate the number of pricks administered to leaves. DW is the non-template negative control. RT-LAMP reactions were conducted at 63 °C for 60 min.

Finally, we compared detection times for the toothpick sampling methods using a 10-fold dilution series of purified RNA from the same leaf. The detection times for the one- and three-prick methods were approximately 21–22 and 20–21 min, almost corresponding to the peaks for 10 and 100 pg of purified RNA, respectively, which were >100-fold higher than the detection limit of 100 fg (Figure 6). This result indicates that the toothpick sampling method collects sufficient ToMMV for detection by RT-LAMP, even using the single-prick method.

## 4. Discussion

Constant outbreaks of emerging viruses have indicated the urgency of controlling viruses to maintain a stable food supply. Tomatoes are among the most widely produced vegetables, and tomato plants are infected by many viruses, satellite viruses, and viroids, including 45 novel species discovered this decade [3]. Although the identification of causal pathogens is critical for appropriate disease management, it is difficult to achieve based on symptom observation alone because the symptoms of various viruses are often similar. Therefore, it is necessary to establish accurate detection methods for each virus. In the present study, we developed the first RT-LAMP-based method for sensitive and specific detection of ToMMV. The designed primer set showed 10-fold higher sensitivity than conventional ToMMV-specific RT-PCR (Figure 4B), and high specificity with no cross-reactivity for four closely related tobamoviruses (TMV, ToMV, ToBRFV, and RheMV) (Figure 3 and Figure 5A,C), and PaMMV, which was reported to cross-react with ToMMV [42] (Figure 3). The high specificity of LAMP is theoretically based on the use of four to six different primers that independently target six to eight regions on the target sequence [43]. Although mixing multiple primers generally results in primer dimers and false positives [44], we also tested a non-template control and verified that no false positives occurred.

To facilitate and shorten the detection process, we adopted a toothpick sampling method that allowed the successful detection of ToMMV from infected tomato leaves within 30 min. This method eliminates the need for expensive equipment and laborious RNA purification, is based on easily accessible and inexpensive toothpicks, requires no expertise, and generates no organic solvent effluents. These features allow for on-site detection and reduce the labor associated with leaf sampling. The disposability of toothpicks also avoids problems such as carryover contamination caused by target nucleic acid adhering to experimental instruments. Therefore, the proposed method can easily be applied to point-of-care testing of ToMMV infection in the field, as have methods for other tomato viruses such as tomato yellow leaf curl virus, tomato chlorosis virus, and tomato infectious chlorosis virus [5,45].

To ensure a practical and reliable diagnosis, it is also important to optimize the prick site based on virus localization. Previous studies on TMV have indicated that tobamoviruses initially accumulate in roots, subsequently move to uppermost leaves, and then spread systemically [46,47]. Therefore, sampling the uppermost leaves is appropriate for diagnosis in the early infection stage, especially when symptoms are not yet evident. In our study, viral symptoms such as mottling, necrosis, and distortion appeared on tomato plants several weeks after ToMMV inoculation (Figure S2), but positive results were obtained from the uppermost leaves of asymptomatic plants just 1 week after inoculation (Figure 5A,C). Thus, the developed RT-LAMP method facilitates the detection of the virus even before symptoms appear, therefore preventing its spread. In cases where plants grow high and sampling the uppermost leaves is difficult, it may be necessary to diagnose other parts of the plants. Within the plant body, viruses are transported from source to sink organs via the phloem, along with photosynthates [48,49]. The strongest sink organs vary with the plant growth stage; in the reproductive stage, these are the fruit and seeds [50,51]. Therefore, it may be worthwhile to determine whether fruit and seeds are suitable target sites for LAMP testing.

Previous studies have developed ToMMV detection methods based on RT-PCR [15,52] and real-time RT-PCR [42]. These methods involve sequence analysis of amplified products, which provide valuable information on the lineages, mutations, and other relevant genetic features of viruses. However, these methods are not suitable for rapid on-site diagnosis due to the need for specialized equipment and long detection times. By contrast, the developed RT-LAMP method cannot differentiate between ToMMV sequences but offers the rapid and sensitive diagnosis required for field application. Therefore, appropriate detection methods should be selected according to specific applications.

LAMP-based virus detection is practical for the control of viral diseases and for epidemiological research, including field studies of virus occurrence, local distribution, host range identification, and vector discovery. At present, only a handful of plants (tomato, pepper, chickpea, eggplant, and pea) have been reported as natural hosts for ToMMV [12,17,26,27,28]. However, considering the broad host range of TMV [53], ToMMV may be able to infect many more plant species [15], including important crops and weeds around farms that may act as virus reservoirs. Some sequences reported as ToMV in GenBank correspond to ToMMV [19], which suggests that the distribution of ToMMV may be much broader than is currently thought. Investigating the status of outbreak areas and host ranges will provide practical evidence for developing control strategies for ToMMV, and our RT-LAMP method is anticipated to contribute to this end. Seed-borne spread of ToMMV is becoming a concern under the increasing global seed trade and is mainly monitored using RT-PCR-based assays [54]. The proposed rapid, highly sensitive RT-LAMP detection method has the potential to improve the accuracy and efficiency of ToMMV quarantine measures.

## Figures and Tables

**Table 1:** Primers used for RT-LAMP and RT-PCR in this study.

Primer Name	Sequence (5′–3′)	Length (nt)	Position *	Reference
ToMMV_2-3_FOP	ACTAATGAAAGAAAAGGGCGG	21	5585–5605 (F3)	This study
ToMMV_2-3_BOP	ATATTTATTAATTCTACAGGGTCG	24	5798–5775 (B3)	This study
ToMMV_2-3_FIP (F1c ** + F2)	CGTCTCGGTATCATCTTCAATCAAA	47	5689–5665 (F1c)	This study
	TCTAATTTCCGTAAGAAACAAG		5606–5627 (F2)	
ToMMV_2-3_BIP (B1c ** + B2)	CAGTCGCGGGATCTGATTCG	40	5691–5710 (B1c)	This study
	ATGCTGATGATAAAAACACG		5770–5751 (B2)	
ToMMV_2_LF	TCACTAACTCCATAACTCTCCTGGT	25	5652–5628 (LF)	This study
ToMMV_2_LB	ATGTCTTACGCTATTACTTCTCCGT	25	5719–5743 (LB)	This study
Tob-Uni1	ATTTAAGTGGASGGAAAAVCACT	21	6285–6263	[38]
Tob-Uni2	GTYGTTGATGAGTTCRTGGA	18	5486–5505	
ToMMV-F	CGACCCTGTAGAATTAATAAATATT	25	5775–5799	[15]
ToMMV-R	CACTCTGCGAGTGGCATCCAAT	22	6063–6042	

* Genome position refers to the nucleotide sequence of Tomato mottle mosaic virus (ToMMV) isolate MX5 (GenBank accession no. KF477193). ** F1c and B1c indicate the complementary sequences of F1 and B1.

## Data Availability

All data produced in the present study are contained in the manuscript.

## Supplementary Materials

The following supporting information can be downloaded at: https://www.mdpi.com/article/10.3390/v15081688/s1, Table S1: Tobamoviruses used for primer design and the experiments in this study; Figure S1: Direct detection of Tomato mottle mosaic virus (ToMMV) from infected tomato leaves using a wooden toothpick; Figure S2: Symptoms of tomato plants (cultivar “Roma VF”) inoculated with ToMMV.
