# Supplementary material for: A Reverse-Transcription Loop-Mediated Isothermal Amplification Technique to Detect Tomato Mottle Mosaic Virus, an Emerging Tobamovirus
Source: Viruses. 2023 Aug 3;15(8):1688. doi: 10.3390/v15081688 (PMC10459350; doi:10.3390/v15081688)
Supplement: Supplementary file 1 [file viruses-15-01688-s001.zip › Figures S1¿CS2.pptx]

## Slide 1
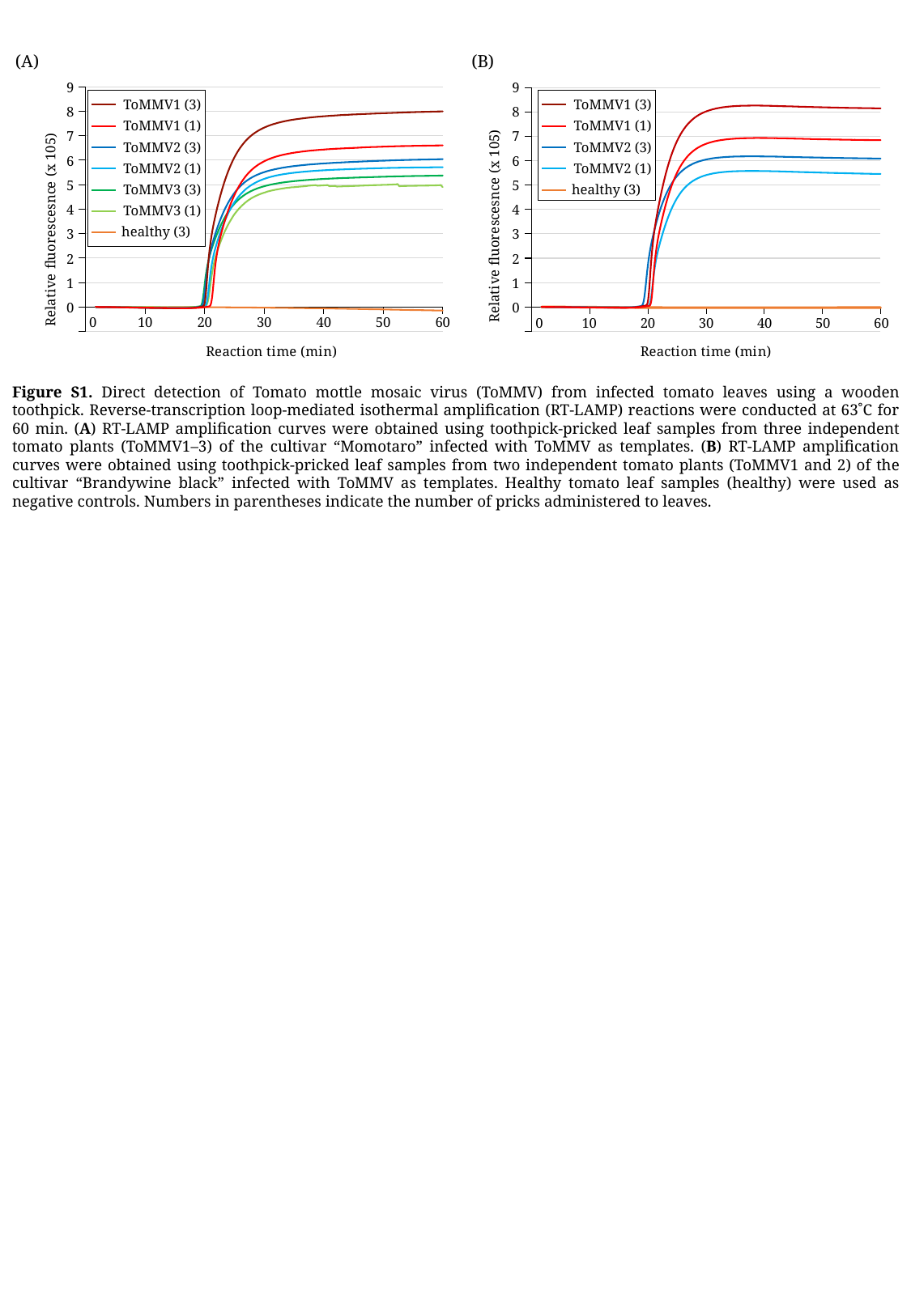

(B)
(A)
9
9
### Chart
| Category | momo1_1 | momo1_3 | momo2_1 | momo2_3 | momo3_1 | momo3_3 | |
|---|---|---|---|---|---|---|---|
### Chart
| Category | ToMMV1(1) | ToMMV1(3) | ToMMV2(1) | ToMMV2(3) | healthy |
|---|---|---|---|---|---|
ToMMV1 (3)
ToMMV1 (1)
ToMMV2 (3)
ToMMV2 (1)
ToMMV3 (3)
ToMMV3 (1)
healthy (3)
ToMMV1 (3)
ToMMV1 (1)
ToMMV2 (3)
ToMMV2 (1)
healthy (3)
8
8
7
7
6
6
5
5
4
4
3
3
2
2
1
1
0
0
0
10
20
30
40
50
60
0
10
20
30
40
50
60
Figure S1. Direct detection of Tomato mottle mosaic virus (ToMMV) from infected tomato leaves using a wooden toothpick. Reverse-transcription loop-mediated isothermal amplification (RT-LAMP) reactions were conducted at 63˚C for 60 min. (A) RT-LAMP amplification curves were obtained using toothpick-pricked leaf samples from three independent tomato plants (ToMMV1–3) of the cultivar “Momotaro” infected with ToMMV as templates. (B) RT-LAMP amplification curves were obtained using toothpick-pricked leaf samples from two independent tomato plants (ToMMV1 and 2) of the cultivar “Brandywine black” infected with ToMMV as templates. Healthy tomato leaf samples (healthy) were used as negative controls. Numbers in parentheses indicate the number of pricks administered to leaves.

## Slide 2
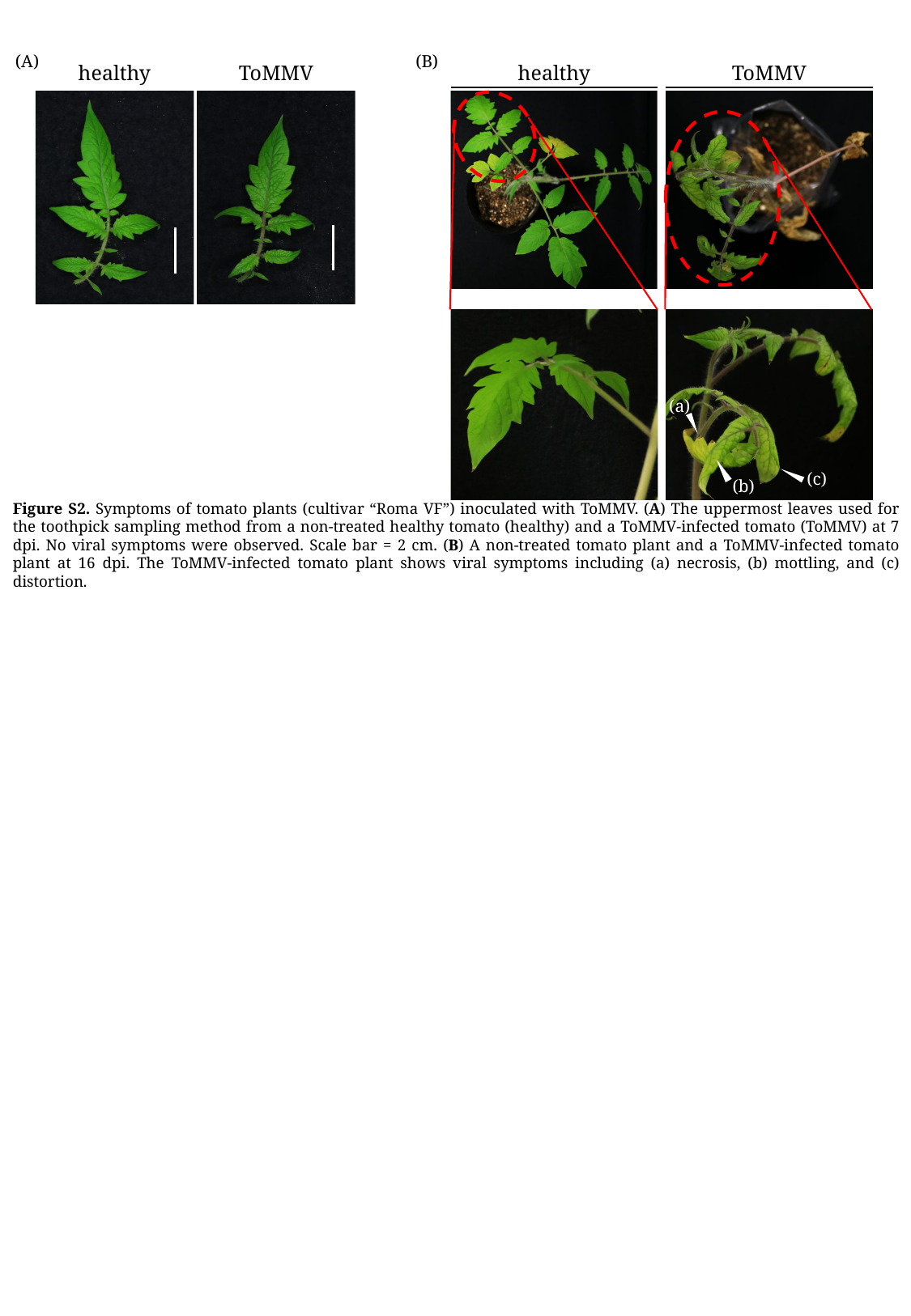

(A)
(B)
healthy
ToMMV
healthy
ToMMV
(a)
(c)
(b)
Figure S2. Symptoms of tomato plants (cultivar “Roma VF”) inoculated with ToMMV. (A) The uppermost leaves used for the toothpick sampling method from a non-treated healthy tomato (healthy) and a ToMMV-infected tomato (ToMMV) at 7 dpi. No viral symptoms were observed. Scale bar = 2 cm. (B) A non-treated tomato plant and a ToMMV-infected tomato plant at 16 dpi. The ToMMV-infected tomato plant shows viral symptoms including (a) necrosis, (b) mottling, and (c) distortion.
