# Supplementary material for: A Reverse-Transcription Loop-Mediated Isothermal Amplification Technique to Detect Tomato Mottle Mosaic Virus, an Emerging Tobamovirus
Source: Viruses. 2023 Aug 3;15(8):1688. doi: 10.3390/v15081688 (PMC10459350; doi:10.3390/v15081688)
Supplement: Supplementary file 1 [file viruses-15-01688-s001.zip › Table S1.pdf]

Table S1. Tobamoviruses used for primer design and the experiments in this study.

Species	Isolate name	Origin	GenBank accession no.
tomato mottle mosaic virus	MX5	Mexico	KF477193
	12-515LLE-1	USA	KM000123
	10-100	USA	KP202857
	YYMLJ	China	KR824950
	TiLhaLJ	China	KR824951
	NY-13	USA	KT810183
	VLC-1	Spain	KU594507
	SC13-05	USA	KX898033
	CA16-01	USA	KX898034
	Hainan	China	MG171192
	Hn18	China	MG920804
	Hn19	China	MG920805
	Hn23	China	MG920806
	CpB1	Brazil	MH128145
	HN	China	MH381817
	19-02305	Netherlands	MN654021
	LN	China	MN853592
	Shandong	China	MW373515
	SY11026	unknown	MW441234
	DSMZ PV-1267	USA	MW582804
	NVWA5785660	Netherlands	MZ713255
	NVWA36783860	China	MZ713256
	NVWA41106813	China	MZ713257
	GD-2020	China	OK180812
	ToMMV_83	Viet Nam	OK334224
	DSMZ PV-1342	Mauritius	ON013925
	MZWD	China	ON146334
	NPPO-NL 41979470	Netherlands	ON987482
	NPPO-NL 41979438	France	ON987481
	NPPO-NL 41979411	Spain	ON987480
tobacco mosaic virus	Variant1	unknown	V01408
	OM	Japan	D78608
tomato mosaic virus	Queensland	Australia	AF332868
	L	Japan	X02144
tomato brown rugose fruit virus	Tom1-Jo	Jordan	KT383474
	DSMZ PV-1241	Israel	MZ202349

rehmannia mosaic virus	Henan	China	EF375551
	Japanese	Japan	AB628188
paprika mild mottle virus	Japanese	Japan	AB089381
